# Effect of nonsteroidal anti-inflammatory drugs on *β*-catenin protein levels and catenin-related transcription in human colorectal cancer cells

**DOI:** 10.1038/sj.bjc.6601901

**Published:** 2004-06-08

**Authors:** S H Gardner, G Hawcroft, M A Hull

**Affiliations:** 1 Molecular Medicine Unit, University of Leeds, St James's University Hospital, Leeds LS9 7TF, UK

**Keywords:** *β*-catenin, colorectal cancer, cyclin D1, nonsteroidal anti-inflammatory drug

## Abstract

Elevated *β*-catenin levels in human colorectal cancer (CRC) cells lead to increased *trans*-activation of ‘protumorigenic’ *β*-catenin/T-cell factor (TCF) target genes such as *cyclin D1*. Therefore, possible targets for the anti-CRC activity of nonsteroidal anti-inflammatory drugs (NSAIDs) are *β*-catenin and catenin-related transcription (CRT). We tested the antiproliferative activity and the effects on levels of *β*-catenin and cyclin D1 protein, as well as CRT (measured using a synthetic *β*-catenin/TCF-reporter gene [TOPflash]), of a panel of NSAIDs (indomethacin, diclofenac, sulindac sulphide and sulphone, rofecoxib; range 10–600 μM) on SW480 human CRC cells *in vitro*. Following NSAID treatment, there was no consistent relationship between reduced cell proliferation, induction of apoptosis and changes in *β*-catenin protein levels or CRT. All the NSAIDs, except rofecoxib, decreased nuclear *β*-catenin content and cyclin D1 protein levels in parallel with their antiproliferative activity. However, cyclin D1 downregulation occurred prior to a decrease in total *β*-catenin protein levels and there was no correlation with changes in CRT, suggesting the existence of CRT-independent effects of NSAIDs on *cyclin D1* expression. In summary, NSAIDs have differential effects on *β*-catenin protein and CRT, which are unlikely to fully explain their effects on cyclin D1 and their antiproliferative activity on human CRC cells *in vitro*.

Dysregulation of catenin-related transcription (CRT) is a common early event in intestinal epithelial cells during human colorectal carcinogenesis (Wong and Pignatelli, 2002). Loss of both alleles of the tumour suppressor gene *Adenomatous Polyposis Coli* (*APC*) is believed to occur prior to development of the benign precursor lesion of colorectal cancer (CRC), the colorectal adenoma (or polyp), in the majority of cases of sporadic colorectal carcinogenesis (Wong and Pignatelli, 2002). Loss of APC function (or less commonly ‘gain-of-function’ mutation of the *β*-catenin gene *CTNNB1*) leads to decreased *β*-catenin degradation and a subsequent increase in cytoplasmic and nuclear *β*-catenin protein levels (Herter *et al*, 1999; Wong and Pignatelli, 2002). This, in turn, leads to increased CRT via formation of a transcriptional complex with T-cell factor (TCF)/lymphoid enhancer factor transcription factors and subsequent *trans*-activation of several *β*-catenin/TCF target genes such as *cyclin D1*, *peroxisome proliferator-activated receptor δ*, *matrix metalloproteinase-7* and *c-MYC*, which are all understood to play a role in colorectal carcinogenesis (Wong and Pignatelli, 2002). Therefore, dysregulated CRT is a potential target for chemoprevention and treatment of CRC.

We, and others, have previously demonstrated that the nonsteroidal anti-inflammatory drug (NSAID) indomethacin decreases *β*-catenin protein levels in human CRC cells *in vitro* and in neoplastic epithelial cells of dimethyl-hydrazine-induced murine colonic tumours *in vivo* (Smith *et al*, 2000; Brown *et al*, 2001a; Hawcroft *et al*, 2002). In addition, Dihlmann *et al* (2001) have reported that indomethacin decreases CRT (measured using the *β*-catenin/TCF-reporter gene TOPflash) in human CRC cells. However, it remains unclear whether modulation of *β*-catenin expression and/or CRT is a property shared by other NSAIDs. In our initial study, downregulation of *β*-catenin protein was not evident in human CRC cells treated with aspirin or the experimental selective cyclooxygenase (COX)-2 inhibitor NS-398 (Smith *et al*, 2000). By contrast, it has been reported that treatment with sulphide and sulphone metabolites of the NSAID sulindac is associated with decreased *β*-catenin protein levels in human CRC cells (Thompson *et al*, 2000; Li *et al*, 2002; Rice *et al*, 2003). Dihlmann *et al* (2001) have also shown that aspirin decreases CRT, as well as indomethacin.

No previous study has systematically investigated a series of NSAIDs in order to determine the relevance of changes in *β*-catenin levels and/or CRT to the well-characterised antiproliferative activity of these drugs on human CRC cells *in vitro*. Therefore, we tested the effects of a diverse panel of NSAIDs, with differing selectivity for the two COX isoforms (Table 1

**Table 1:** The COX isoform selectivity of the NSAIDs used

**Drug^a^**	**COX-1 IC_50_ (*μ*M)^b^**	**COX-2 IC_50_ (*μ*M)^b^**	**COX selectivity**
Indomethacin	0.013	0.13	COX-1>COX-2
Diclofenac	0.075	0.02	COX-2>COX-1
Sulindac sulphide	1.9	1.21	COX-1=COX-2
Sulindac sulphone^c^	N/A	N/A	N/A
Rofecoxib	63	0.31	COX-2>>COX-1

a Indomethacin was included as a positive control as it has antineoplastic activity and decreases *β*-catenin protein levels in intestinal epithelial cells *in vitro* and *in vivo* (Smith *et al*, 2000; Brown *et al*, 2001a). The chemically related prodrug sulindac is converted *in vivo* to a sulphide or, to a lesser extent, a sulphone metabolite, which have both been demonstrated to possess anti-CRC activity *in vitro* and *in vivo* (Piazza *et al*, 1997a, b; McEntee *et al*, 1999; Brown *et al*, 2001b). Diclofenac was included as it is a widely used, well tolerated drug, for which there is only scanty evidence for anti-CRC activity (Hixson *et al*, 1994). Rofecoxib possesses high selectivity for COX-2 and so may find increased acceptability as a possible chemoprevention agent due to improved upper gastrointestinal tolerability (Bombardier *et al*, 2000).

b The precise IC_50_ value for COX-1 and COX-2 inhibition by individual NSAIDs is dependent on the assay used but the overall COX isoform selectivity of each NSAID is usually consistent between methodologies. The IC_50_ values quoted are from the large study by Warner *et al* (1999).

c Sulindac sulphone has no COX inhibitory activity.

), on the expression of *β*-catenin protein, cyclin D1 protein and CRT by SW480 human CRC cells and correlated these changes with the antiproliferative activity of each NSAID.

## MATERIALS AND METHODS

### Cell culture

SW480 human sporadic CRC cells (European Collection of Animal Cell Cultures, Porton Down, UK) were grown in RPMI 1640 medium plus GlutaMAX-I™ supplemented with 10% (v/v) foetal bovine serum (FBS), 1000 U ml^−1^ penicillin and 500 U ml^−1^ streptomycin (all Invitrogen, Paisley, UK). SW480 human CRC cells were cultured on tissue culture plastic at 37°C, in the presence of 5% CO_2_. Cells were routinely subcultured using 0.25% (w v^−1^) trypsin/1 mM ethylenediaminetetraacetic acid (EDTA) solution (Invitrogen). Cells were counted using a haemocytometer and viability was assessed by exclusion of 0.4% (w v^−1^) Trypan blue (Sigma, Poole, UK). 1 × 10^5^ viable cells were seeded per 35 mm well and were incubated for 96 h before NSAID treatment was commenced.

### Drugs and antibodies

Indomethacin and diclofenac were obtained from Sigma. Sulindac sulphide and sulindac sulphone were obtained from ICN Biomedicals, Basingstoke, UK. For some experiments, sulindac sulphide was also obtained from Sigma. Rofecoxib powder was a kind gift from Merck Sharp & Dohme Ltd., Hoddesdon, Herts., UK. A 100 mM stock solution of diclofenac was prepared in sterile distilled water. The other drugs were prepared as 100 mM stock solutions in dimethyl sulphoxide (DMSO; Sigma). Control cultures contained either DMSO or distilled water at an equivalent (v v^−1^) dilution to that used for the highest concentration of each NSAID.

Mouse monoclonal anti-chicken *β*-catenin antibody (6F9) and mouse monoclonal anti-human *β*-actin antibody (AC15) were obtained from Sigma. Mouse monoclonal anti-human cyclin D1 (A12) was obtained from Santa Cruz Biotechnology (Santa Cruz, CA, USA). Horseradish peroxidase-conjugated rabbit anti-mouse IgG was obtained from DakoCytomation (Ely, UK).

### Cell counting assays

For each experiment, nonadherent and adherent cells in three individual wells were counted after a 96 h culture period (denoted time 0 h). Old medium was removed from the other wells and fresh medium containing a known concentration of NSAID or solvent carrier alone, was added to triplicate wells and incubated for 24, 48 or 72 h. At each time point, medium containing nonadherent cells was aspirated from triplicate wells and centrifuged for 5 min at 200 ***g***, prior to resuspension in a small volume of medium for counting. Adherent cells were then harvested by trypsinisation and counted as above. Data are represented as the mean and standard error of the mean (s.e.m.) cell count (*n*=3), for each time point and drug concentration.

### Indirect immunofluorescence

SW480 human CRC cells were grown on glass coverslips in 35 mm wells as described above. After NSAID treatment for 48 h, indirect immunofluorescence for *β*-catenin was performed on adherent cells on coverslips as described (Smith *et al*, 2000).

### Fluorescence microscopy of nonadherent SW480 human CRC cells

Nonadherent SW480 human CRC cells present in medium after 48 h NSAID treatment were aspirated, pelleted and resuspended in 4% (w v^−1^) paraformaldehyde in phosphate-buffered saline (PBS). Fixed cells were resuspended in PBS containing 0.5 *μ*g ml^−1^ Hoechst 33258 (ICN Biomedicals). The percentage number of morphologically apoptotic cells was counted by fluorescence microscopy as described (Ko *et al*, 2002).

### Measurement of caspase-3/-7 activity

SW480 human CRC cells (3.4 × 10^3^). were placed in individual wells of a flat-bottomed 96-well plate and cultured for 96 h in order to mirror the experimental protocol used for cell counting assays. Cells were treated with NSAIDs or an equivalent dilution of carrier solvent for 48 h before addition of caspase substrate. Caspase-3/-7 activity was measured at 90 min by a fluorescence plate reader (FLUOstar Galaxy, Isogen Life Science, Maarssen, The Netherlands) as per the manufacturer's instructions (Apo-ONE™ homogeneous caspase-3/-7 assay, Promega UK Ltd., Southampton, UK). Data are presented as the mean (+s.e.m.) percentage fluorescence value for each NSAID compared with the appropriate carrier control (*n*=3). None of the NSAIDs had any direct activity on the caspase substrate in ‘no cells’ control wells (data not shown).

### Western blot analysis

Adherent cells were lysed in 50 mM Tris-HCl (Melford Labs. Ltd., Ipswich, UK) buffer (pH 7.2) containing 0.137 M sodium chloride (BDH, Poole, UK), 1% Brij 96 (Fluka, Gillingham, UK), 1 mM EDTA and Complete™ protease inhibitor cocktail (Roche Diagnostics, Germany). Whole cell lysates were prepared as described (Hawcroft *et al*, 2002) and the total protein concentration was determined using a BioRad DC kit (BioRad, Hemel Hempstead, UK). Sodium dodecyl–sulphate polyacrylamide gel electrophoresis (SDS–PAGE) was performed on 20 *μ*g total protein aliquots, alongside MagicMark Western Standards (Invitrogen, Paisley, UK), using 10% NuPAGE Bis-Tris 1.0 mm gels (Invitrogen, Paisley, UK) as described (Hawcroft *et al*, 2003). Proteins were transferred to Hybond P polyvinylidene fluoride membranes (Amersham Pharmacia Biotech, Amersham, UK) by wet blotting. Membranes were blocked with 5% (w v^−1^) dried skimmed milk powder in PBS (blocking solution) containing 0.05% (v v^−1^) Tween 20 (Sigma) for 1 h at 20°C. Membranes were probed with primary antibodies against *β*-catenin (1/5000 dilution) or *β*-actin (1/2500 dilution) in blocking solution for 1 h at 20°C. Anti-cyclin D1 antibody (1/500 in blocking solution) was applied overnight at 4°C. Horseradish peroxidase-conjugated secondary antibody (1/5000 or 1/1000 [for cyclin D1] dilution) in blocking solution was incubated with the membrane for 1 h at 20°C. Immunoreactive protein was detected using ECL chemiluminescence (Pierce, Chester, UK).

### Densitometric analysis

A Bio-Rad Molecular Imager FX was used to quantify individual bands. The background intensity was subtracted and the bands were quantified using Quantity One software (Bio-Rad). The relative intensity of the *β*-catenin band to the *β*-actin band was determined. Each *β*-catenin/*β*-actin ratio was then expressed as a percentage of the control *β*-catenin/*β*-actin ratio (which was given an arbitrary value of 100%). Data are represented as the mean (and s.e.m.) percentage of the control *β*-catenin/*β*-actin ratio value (*n*=3), for each time point and drug treatment.

### Transient DNA transfection and dual-luciferase reporter assays

Experiments on the effect of NSAIDs on CRT, using the *β*-catenin/TCF-reporter construct TOPflash (Korinek *et al*, 1997), were performed in an identical manner to that described for the cell proliferation and apoptosis assays, except that transient DNA transfection of cells occurred during a 3 h period prior to addition of drug or carrier control. GeneJuice™ transfection reagent (Novagen, Madison, WI, USA) was added to serum-free RPMI 1640 medium plus GlutaMAX-I™ and incubated for 5 min before addition of the appropriate plasmid DNA (TOPflash or FOPflash (1 *μ*g; both Upstate Biotechnology, Lake Placid, NY, USA) with pRL-TK (0.5 *μ*g; Promega). The GeneJuice™-DNA mix was incubated for 30 min at 20°C before incubation with cells for 3 h at 37°C. The transfection medium was removed and fresh culture medium, containing drug or carrier control, was added. After incubation for 48 h at 37°C, cells were washed once with PBS, lysed with Passive Lysis Buffer and Dual-Luciferase reporter assays (both Promega) were performed as per the manufacturer's instructions. *Firefly* (TOPflash or FOPflash) luciferase activity was corrected for *Renilla* luciferase activity (pRL-TK) to control for transfection efficiency. In order to exclude any CRT-independent effect on thymidine kinase (tk) minimal promoter activity of TOPflash, TOPflash activity was normalised to the ratio of the FOPflash (which contains an identical tk promoter) activity between NSAID-treated and control cells, termed the FOPflash ratio. Data are expressed as the mean (and s.e.m.) of triplicate values of the normalised TOPflash activity, with the corresponding drug-treated/control cell FOPflash activity ratio.

### Statistical analysis

One-way analysis of variance (ANOVA) was used to determine the significance of the effect of different concentrations of NSAIDs on cell number, the *β*-catenin/*β*-actin protein ratio and normalised TOPflash activity. The Bonferroni test was used for *post hoc* comparison of the effects of particular NSAID concentrations compared with control cell cultures. Statistical significance was assumed if the *P*-value was less than or equal to 0.05.

## RESULTS

### Effect of NSAIDs on SW480 human CRC cell proliferation and apoptosis

Our previous study demonstrated that indomethacin treatment induced G1 arrest and apoptosis (measured by flow cytometric DNA content analysis), as well as decreased *β*-catenin protein levels, in four human CRC cell lines, regardless of the presence of mutant *APC* alleles or COX-2 expression (Smith *et al*, 2000). Therefore, we used only one human CRC cell line for the current study. We chose SW480 human CRC cells as they contain no functional APC and hence have high basal CRT and *cyclin D1* expression (Shtutman *et al*, 1999; Hawcroft *et al*, 2002). In addition, SW480 human CRC cells do not express COX-2 (Smith *et al*, 2000) and therefore reflect the COX expression pattern of intestinal epithelial cells at early stages of colorectal carcinogenesis, of most relevance to CRC chemoprevention (Chapple *et al*, 2000).

**Figure 1:**
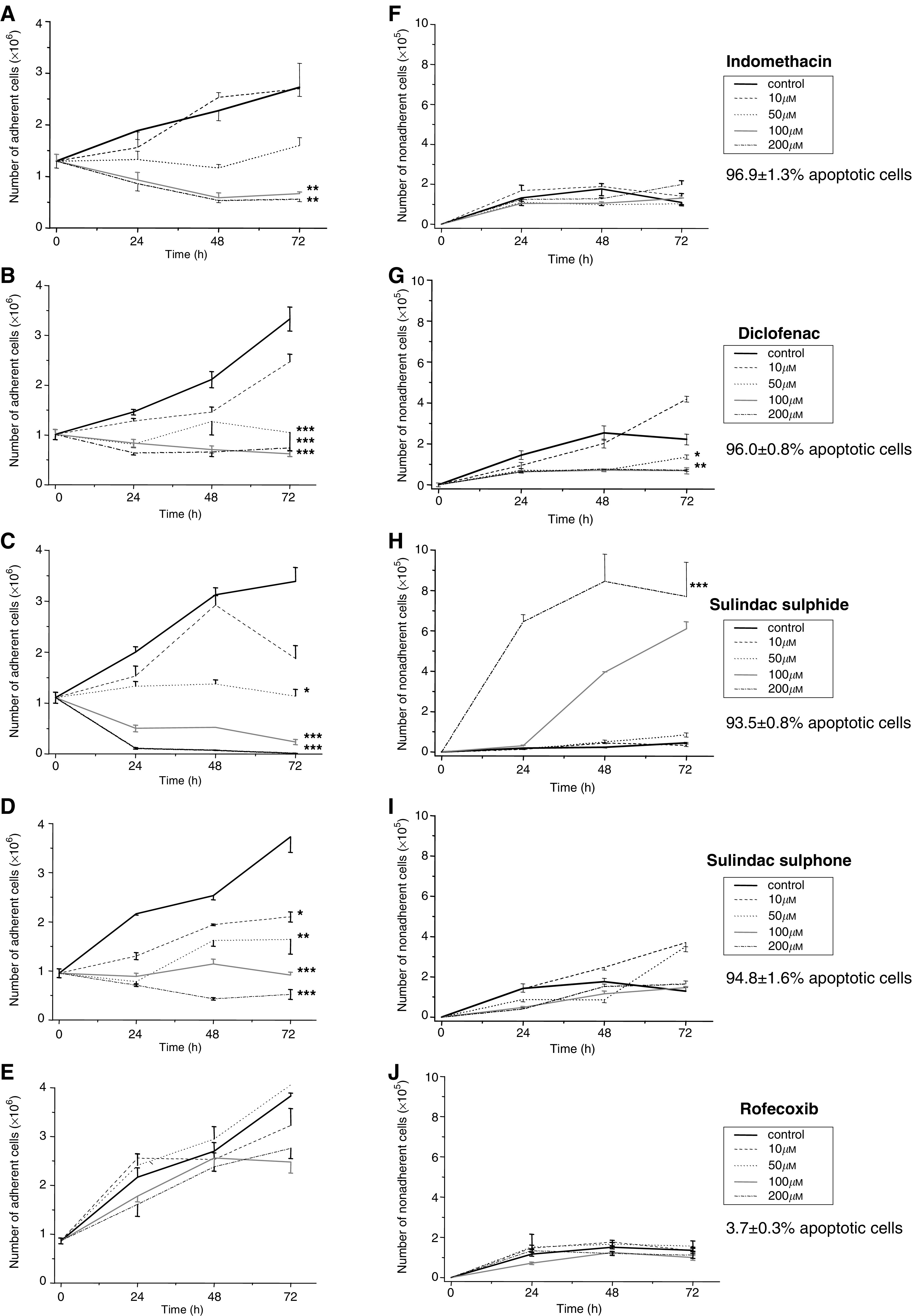
Effect of NSAIDs on SW480 human CRC cell proliferation and apoptosis. The number of adherent (viable) cells and nonadherent (apoptotic) cells were counted at 24–72 h time points. Data are expressed as the mean and s.e.m. number of adherent cells (× 10^6^; parts **A–E**) and nonadherent cells (× 10^5^; parts **F–J**). For ease of analysis, error bars are sometimes unidirectional. ^*^ *P*<0.05, ^**^ *P*<0.01, ^***^ *P*<0.001 for all time points for a given concentration of a NSAID compared with the carrier control (one-way ANOVA with *post hoc* Bonferroni test). Parts **F–J** include the mean (±s.e.m.) percentage number of Hoechst 33258-stained, morphologically apoptotic nonadherent cells after 48 h treatment with the highest concentration of NSAID (*n*=3).

The viability of adherent SW480 human CRC cells was consistently greater than 95%. Therefore, the number of adherent cells at each time point was used to measure net cell proliferation. We, and others, have previously demonstrated that, following NSAID treatment of human CRC cells, the number of nonadherent cells accurately reflects the degree of apoptosis that has occurred (Shiff *et al*, 1996; Piazza *et al*, 1997b; Qiao *et al*, 1997; Smith *et al*, 2000). In the experiments described herein, the nonadherent cell population consisted of greater than 90% morphologically apoptotic cells following treatment with all the NSAIDs except rofecoxib (Figure 1F–J

). Therefore, the number of nonadherent SW480 human CRC cells, at each time point, was used as a measure of apoptosis of NSAID-treated SW480 human CRC cells.

There was a 2–3-fold increase in the number of adherent SW480 human CRC cells over 72 h in the presence of solvent carrier alone, in all experiments (Figure 1A–E). In the absence of drug treatment, there was only a small number of morphologically nonapoptotic, nonadherent cells (approximately 1 × 10^5^ cells/24 h), which did not increase during the 72 h experiment (Figure 1F–J).

**Figure 2:**
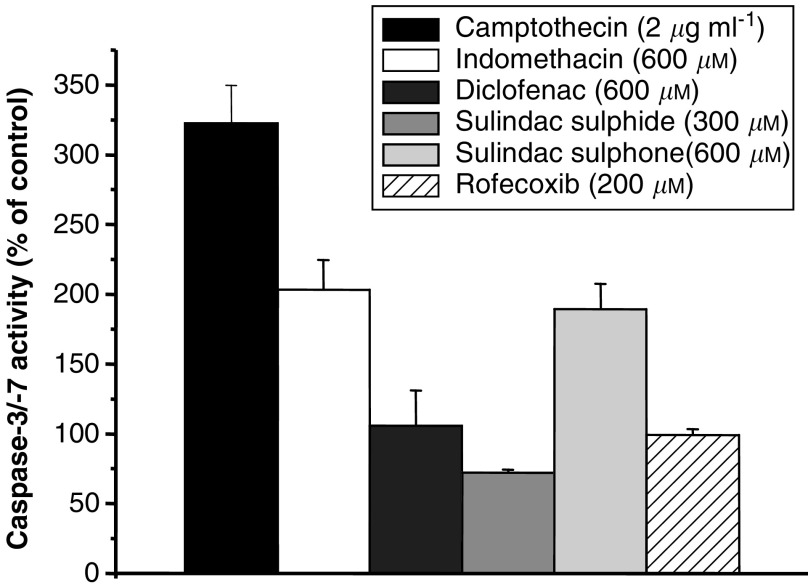
Effect of NSAIDs on caspase-3/-7 activity in SW480 human CRC cells. Caspase-3/-7 substrate was added after 48 h incubation of SW480 human CRC cells with NSAIDs. Generation of fluorescent product (activation 485 nm, emission 520 nm) by activated caspases is presented as the mean (+s.e.m.) percentage of the fluorescence value of cells treated with an identical dilution of solvent carrier only (*n*=3). Treatment with the DNA topoisomerase I inhibitor camptothecin (2 *μ*g ml^−1^ for 48 h) was used as a positive control.

We chose to include indomethacin in the panel of NSAIDs tested in this study in order to act as a control which would aid comparison with our previous studies on indomethacin and *β*-catenin (Smith *et al*, 2000; Hawcroft *et al*, 2002). Treatment with indomethacin resulted in a concentration-dependent reduction in proliferation (Figure 1A). The decrease in adherent SW480 human CRC cells was statistically significant at all three time points when indomethacin was present at a concentration equal to or above 400 *μ*M (Figure 1A). Indomethacin treatment was associated with a small increase in the degree of apoptosis of SW480 human CRC cells measured by nonadherent cell counting in this experiment (Figure 1F). However, proapoptotic activity of indomethacin was readily apparent in the caspase-3/-7 activity assay (Figure 2

).

Diclofenac treatment was also associated with a concentration-dependent antiproliferative effect on SW480 human CRC cells, which reached statistical significance at concentrations equal to and above 200 *μ*M (Figure 1B). Unlike indomethacin, the antiproliferative activity of diclofenac associated with concentrations above 200 *μ*M, occurred in conjunction with a significant decrease in apoptosis between 24 and 72 h (Figure 1G). Data from the caspase activity assay were consistent with these findings, in that the highest concentration of diclofenac (600 *μ*M) did not induce caspase activation in SW480 human CRC cells at 48 h compared with control cells (Figure 2).

The two metabolites of sulindac, sulphide and sulphone, both demonstrated concentration-dependent antiproliferative activity against SW480 human CRC cells (Figure 1C–D). However, the approximate IC_50_ value for the antiproliferative effect of sulindac sulphide at 72 h (100 *μ*M) was approximately half that of sulindac sulphone (200 *μ*M). Treatment with sulindac sulphide at concentrations at and above 200 *μ*M was associated with a pronounced increase in apoptosis (Figure 1H), unlike equivalent or higher concentrations of sulindac sulphone (Figure 1I). Interestingly, treatment with 300 *μ*M sulindac sulphide was associated with a reduction (not an increase, unlike the other proapoptotic NSAIDs, including sulindac sulphone) in caspase-3/-7 activity (Figure 2).

Rofecoxib (up to 200 *μ*M) had little effect on the proliferation of SW480 human CRC cells compared with the other NSAIDs that were tested in this study (Figure 1E). There was a small decrease in the adherent cell number at 72 h following incubation with 100 and 200 *μ*M rofecoxib (Figure 1E) but this did not reach statistical significance. No increase in nonadherent SW480 human CRC cells was evident following rofecoxib treatment and only 3.7% of such cells had morphological characteristics of apoptosis (Figure 1J). In addition, rofecoxib did not induce caspase-3/-7 activity in SW480 human CRC cells (Figure 2). We were unable to test rofecoxib at concentrations higher than 200 *μ*M because of poor solubility of the stock solution in aqueous cell culture medium.

### Effect of NSAIDs on *β*-catenin protein levels in SW480 human CRC cells

**Figure 3:**
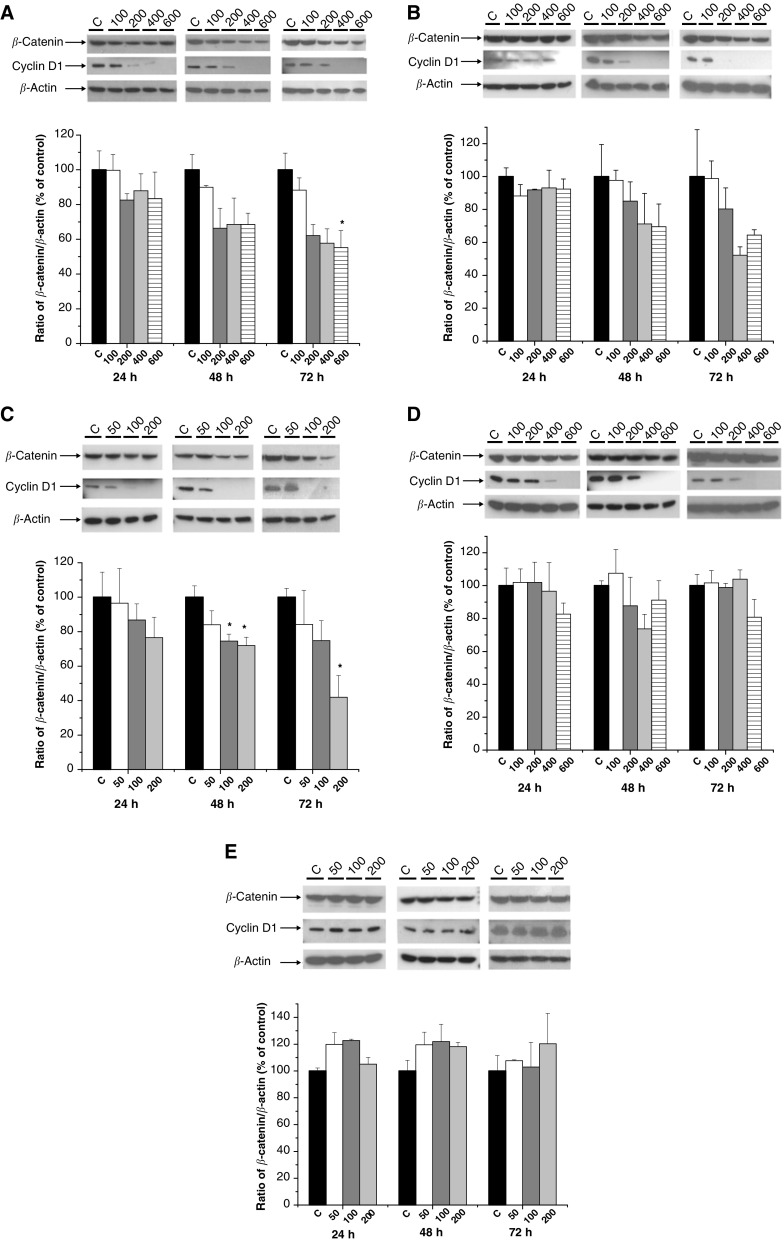
Effect of NSAIDs on *β*-catenin and cyclin D1 protein levels in SW480 human CRC cells. Western blot analysis of *β*-catenin (92 kDa), cyclin D1 (36 kDa) and *β*-actin (42 kDa) protein expression after incubation with differing concentrations (in *μ*M) of NSAIDs. C denotes the carrier control. A representative blot for each NSAID is accompanied by a quantitative analysis of triplicate *β*-catenin blots. Densitometric data are expressed as the mean (+s.e.m.) percentage of the control *β*-catenin/*β*-actin ratio. ^*^P<0.05 for the difference in the *β*-catenin/*β*-actin ratio compared with control cells (one-way ANOVA with *post hoc* Bonferroni test). (**A**) Indomethacin; (**B**) Diclofenac; (**C**) Sulindac sulphide; (**D**) Sulindac sulphone; (**E**) Rofecoxib.

**Figure 4:**
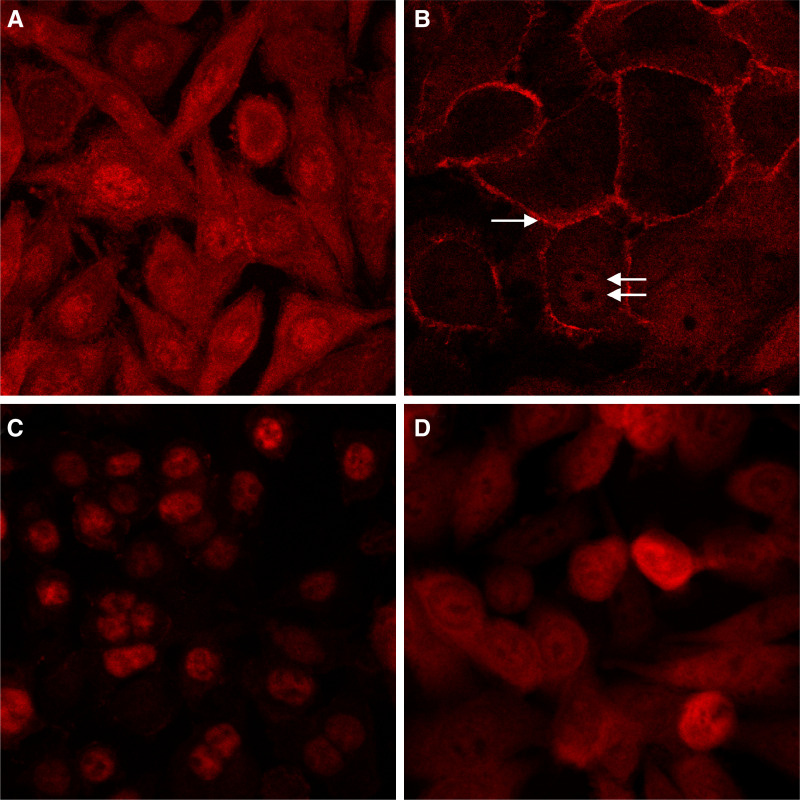
Effect of NSAIDs on *β*-catenin localisation in SW480 human CRC cells. Indirect immunofluorescence for *β*-catenin was performed on adherent cells following 48 h treatment with each NSAID. Confocal microscopy was performed at the same magnification for all cell preparations (× 1000). (**A**) Cells incubated in the presence of 0.6% (v v^−1^) DMSO, (**B**) diclofenac (600 *μ*M)-treated cells demonstrating increased membranous *β*-catenin localisation (arrow) with decreased nuclear (double arrow) and cytoplasmic *β*-catenin intensity (similar changes were apparent following treatment with 600 *μ*M indomethacin and 120 *μ*M sulindac sulphide), (**C**) sulindac sulphide (300 *μ*M)-treated cells exhibiting shrunken nuclei with prominent ‘speckled’ *β*-catenin localisation, (**D**) rofecoxib (200 *μ*M)-treated cells demonstrating a minimal reduction in nuclear *β*-catenin signal.

Indomethacin treatment, at concentrations equal to and above 200 *μ*M, was associated with a time-dependent decrease in *β*-catenin protein levels, which reached statistical significance at 72 h for the 600 *μ*M concentration (Figure 3A

). Similar changes in *β*-catenin protein levels were evident following diclofenac treatment, although these changes did not attain statistical significance (Figure 3B). Diclofenac treatment was associated with a reduction in nuclear and cytoplasmic *β*-catenin immunofluorescence signal, with a corresponding increase in membranous *β*-catenin localisation, as we have previously described for indomethacin (Figure 4B

; Smith *et al*, 2000). Treatment with sulindac sulphide was associated with a time- and concentration-dependent reduction in *β*-catenin protein levels (Figure 3C). There was a 60% reduction in *β*-catenin protein level after 72 h incubation with 200 *μ*M sulindac sulphide which was statistically significant (Figure 3C). Treatment with 120 *μ*M sulindac sulphide for 48 h was associated with a reduction in nuclear *β*-catenin content and increased membranous *β*-catenin localisation in a similar manner to indomethacin and diclofenac (data not shown). However, a higher concentration of sulindac sulphide (300 *μ*M) induced an apparent increase in nuclear *β*-catenin protein content (Figure 4C), which may be explained by nuclear shrinkage producing the false impression of increased *β*-catenin content or selection of ‘resistant’ cells with high nuclear *β*-catenin content. No significant changes in total *β*-catenin protein levels were observed in SW480 CRC cells treated with higher concentrations of sulindac sulphone (Figure 3D). However, treatment with sulindac sulphone (600 *μ*M) was associated with reduced nuclear *β*-catenin protein (data not shown) consistent with a previous report (Rice *et al*, 2003). Rofecoxib treatment had no significant effect on *β*-catenin protein levels or localisation in SW480 human CRC cells (Figures 3E and 4D).

### Effect of NSAIDs on cyclin D1 protein levels in SW480 human CRC cells

Previously, we have demonstrated that indomethacin treatment of SW480 human CRC cells is associated with decreased expression of the *β*-catenin/TCF target gene *cyclin D1* (Smith *et al*, 2000; Hawcroft *et al*, 2002). Therefore, we were interested to determine whether the effects of the NSAIDs on proliferation and apoptosis described above were associated with changes in the cellular content of cyclin D1 protein, which is recognised to play a crucial role in progression through the G1 phase of the cell cycle (Lukas *et al*, 1996). Treatment with all the NSAIDs, except rofecoxib, was associated with a marked decrease in cyclin D1 protein levels, such that there was a complete absence of cyclin D1 protein in SW480 human CRC cells after treatment with high (>200 *μ*M) concentrations of NSAIDs (Figure 3A–E). Therefore, densitometric analysis of cyclin D1 protein levels was not carried out. A decrease in cyclin D1 protein was apparent by 24 h and this effect increased in a concentration-dependent manner. The time and magnitude of the decrease in cyclin D1 protein correlated well with the antiproliferative activity of individual NSAIDs (compare Figures 1 and 3; Table 2

**Table 2:** Summary of the effects of NSAIDs on SW480 human CRC cells

**Drug**	**Anti-proliferative activity^a^**	**Pro-apoptotic activity^a^**	**Effect on total *β*-catenin protein levels^b^**	**Effect on nuclear *β*-catenin content^b^**	**Effect on cyclin D1^b^**	**Effect on CRT^b^, ^c^**
Indomethacin	++	++	↓	↓	↓↓	→ ^*^
Diclofenac	++	+	↓	↓	↓↓	↓
Sulindac sulphide	+++	+++	↓↓	↓^d^	↓↓	↓
Sulindac sulphone	++	++	→	↓	↓	→ ^*^
Rofecoxib	+	−	→	→	→	→ ^*^

a −=none; +=weak; ++=moderate; +++=strong.

b → =No change; ↓=decrease; ↓↓=marked decrease.

c → ^*^Indicates that at least one concentration of the NSAID was associated with an increase in CRT compared with control cells.

d Nuclear *β*-catenin content was reduced after treatment with 120 *μ*M sulindac sulphide for 48 h. A paradoxical increase in nuclear localisation of *β*-catenin was apparent after incubation with 300 *μ*M sulindac sulphide for 48 h.

). However, a simple relationship between alterations in cyclin D1 protein levels and the corresponding change in total *β*-catenin protein levels, associated with individual NSAIDs, was not apparent (Figure 3). For example, a marked decrease in cyclin D1 protein in indomethacin-, diclofenac- and sulindac sulphide-treated cells was associated with quantitatively smaller changes in *β*-catenin protein content, which tended to occur at later time points (Figure 3A–C). Moreover, the abolition of cyclin D1 protein expression in cells treated with high (>400 *μ*M) concentrations of sulindac sulphone did not occur in association with significant changes in *β*-catenin protein content (Figure 3D). In this context, it is perhaps more relevant to compare changes in cyclin D1 protein levels and nuclear *β*-catenin content. There was a good correlation between the reduction in nuclear *β*-catenin content observed by immunofluorescence and the decrease in cyclin D1 protein that was apparent after treatment with any given NSAID (Figures 3 and 4; Table 2)

### Effect of NSAIDs on CRT in SW480 human CRC cells

**Figure 5:**
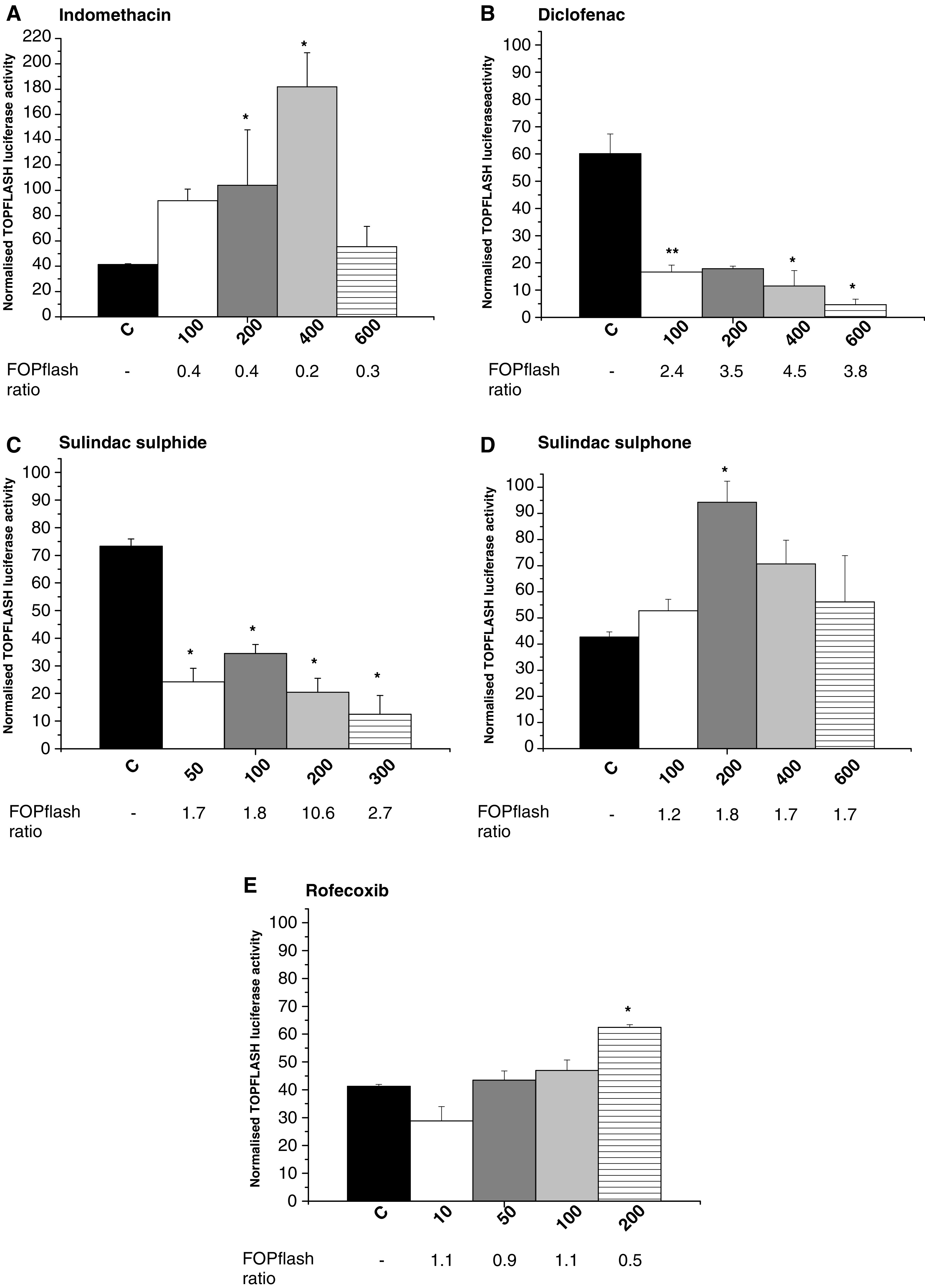
Effect of NSAIDs on CRT in human SW480 CRC cells. Dual-luciferase assays were performed on SW480 human CRC cells transfected with TOPflash or FOPflash vectors, along with *Renilla* pRL-TK plasmid, and then treated for 48 h with differing concentrations (in *μ*M) of NSAIDs. TOPflash and FOPflash activities were corrected for transfection efficiency using the *Renilla* pRL-TK activity. The effect of NSAIDs on TOPflash activity was corrected for CRT-independent effects on the minimal tk promoter downstream of the TCF binding sites by normalisation against the ratio of FOPflash (that contains the identical promoter) activity in drug-treated *vs* control cells (FOPflash ratio). Data from triplicate experiments are expressed as the mean+s.e.m. normalised TOPflash activity for each drug concentration. The corresponding mean FOPflash ratio (*n*=3) is noted below each bar. ^*^P<0.05 for the difference in normalised TOPflash activity compared with control cells (one-way ANOVA with *post hoc* Bonferroni test).

We were then interested to determine the effects of the panel of NSAIDs on CRT in SW480 human CRC cells. Cells were transiently transfected with a synthetic TCF reporter plasmid TOPflash (which consists of three TCF binding sites upstream of a minimal tk promoter and the *Luciferase* open reading frame), prior to treatment with NSAIDs. TOPflash activity in control SW480 human CRC cells was consistently greater than 40-fold higher than the corresponding FOPflash activity, confirming significant basal CRT in cells lacking functional *APC* alleles. In order to exclude nonspecific effects of NSAIDs on gene expression, TOPflash activity in NSAID-treated SW480 human CRC cells was corrected for any drug-induced effects on FOPflash (which is identical to TOPflash except that it contains mutant inactive TCF binding sites). NSAID treatment was associated with numerically small, variable effects on FOPflash activity, which are denoted as the ratio of FOPflash activity in treated *vs* control SW480 human CRC cells (Figure 5

).

Figure 5 (in comparison with Figure 3) demonstrates that there was no consistent relationship between CRT, measured by TOPflash activity, and changes in either total *β*-catenin protein levels, nuclear *β*-catenin localisation or cyclin D1 protein levels in SW480 human CRC cells treated with NSAIDs. CRT was significantly reduced by treatment with diclofenac or sulindac sulphide for 48 h in a concentration-dependent manner, in parallel with reduced *β*-catenin protein levels, although changes in *β*-catenin protein levels associated with the former drug did not reach statistical significance (compare Figures 5B and 3B). Treatment with indomethacin was associated with a biphasic pattern of changes in CRT, such that CRT was increased in the presence of 200–400 *μ*M indomethacin but the increase was abolished at a higher concentration (600 *μ*M) of indomethacin (Figure 5A). Treatment with the two NSAIDs that did not appear to be associated with changes in total *β*-catenin protein levels (sulindac sulphone and rofecoxib) was not associated with reduced CRT (Figure 5D and E). Indeed, certain concentrations of these two agents were actually associated with increased normalised TOPflash activity (Figure 5D and E).

Levels of cyclin D1 protein in SW480 human CRC cells after therapy with different concentrations of diclofenac and sulindac sulphide for 48 h did mirror TOPflash activity but not for indomethacin or sulindac sulphone, which were variably associated with unchanged or increased TOPflash activity despite decreased cyclin D1 protein levels (compare Figures 3 and 5).

## DISCUSSION

This study has revealed that all the NSAIDs tested, except the selective COX-2 inhibitor rofecoxib, have significant antiproliferative activity against SW480 human CRC cells, which is associated with decreased cyclin D1 protein expression (Table 2). Although there was a good correlation between the antiproliferative activity of each NSAID and the time-course and extent of downregulation of the cell cycle regulator *cyclin D1*, the reduction in total *β*-catenin protein levels and changes in CRT demonstrated for individual NSAIDs bore no such relationship (Table 2). However, the reduction in nuclear *β*-catenin content observed by immunofluorescence did parallel changes in cyclin D1 protein levels (Table 2).

SW480 human CRC cells do not express COX-2 (Smith *et al*, 2000). Therefore, this cell line may not be representative of *all* colorectal epithelial cells at early stages of colorectal carcinogenesis (a minority of which contain COX-2 protein; Chapple *et al*, 2000). However, several Groups have not reported differences in the effects of NSAIDs on COX-2-positive compared with COX-2-negative human CRC cell lines (Hixson *et al*, 1994; Yamazaki *et al*, 2002), including changes in *β*-catenin levels (Rice *et al*, 2003), thus justifying the use of SW480 human CRC cells in our experiments.

Although the effects of the NSAIDs used in this study on proliferation and apoptosis of SW480 human CRC cells have been previously described (Hixson *et al*, 1994; Piazza *et al*, 1997b; Smith *et al*, 2000; Yamazaki *et al*, 2002), there has been no previous study of the effects of a series of NSAIDs on expression of *β*-catenin and the cell cycle regulatory protein cyclin D1 by these cells. Therefore, we decided to confirm the data on NSAID-induced changes in SW480 human CRC cell kinetics so that a direct comparison of changes in *β*-catenin and cyclin D1 protein levels could be made with effects on proliferation and apoptosis in the same set of experiments. The concentration range that was used for each NSAID was based on previous data from experiments using human CRC cells (Hixson *et al*, 1994; Piazza *et al*, 1997b; Smith *et al*, 2000; Yamazaki *et al*, 2002). The question of whether the high concentrations of NSAIDs, which are employed in studies using human CRC cells *in vitro*, are relevant to the antineoplastic activity of these agents *in vivo* is currently a subject of debate (Hwang *et al*, 2002; Hull *et al*, 2003). However, the *in vivo* relevance of our current findings on the effect of NSAIDs on *β*-catenin levels and localisation has been confirmed in rodent models (McEntee *et al*, 1999 [sulindac]; Brown *et al*, 2001a [indomethacin, sulindac sulphone]; Oshima *et al*, 2001 [rofecoxib]).

As expected, all the NSAIDs had antiproliferative activity in the 100–600 *μ*M concentration range. The IC_50_ values for the individual NSAIDs were similar to those previously reported (Hixson *et al*, 1994; Piazza *et al*, 1997b; Yamazaki *et al*, 2002). As previously described, rofecoxib had little antiproliferative activity compared with the other NSAIDs that were tested (Yamazaki *et al*, 2002), although the concentration range of this NSAID was restricted by poor solubility. We also confirmed a previous report that the sulphide metabolite of sulindac has more potent antiproliferative activity than sulindac sulphone (Piazza *et al*, 1997b). The effect of the individual NSAIDs on induction of apoptosis was smaller and more variable than the antiproliferative activity. Sulindac sulphide was the only NSAID that induced significant apoptosis (measured by nonadherent cell counting) in parallel with its antiproliferative activity. The small increase in apoptosis associated with the lower concentrations of diclofenac and, to a lesser extent, with sulindac sulphone implies that proapoptotic activity of these NSAIDs may predominate at these concentrations and that proapoptotic and antiproliferative effects of certain NSAIDs may be independent. The decrease in apoptotic cell number associated with higher concentrations of diclofenac may represent a reduction in apoptosis, which would normally occur at cell confluency, rather than a direct NSAID-induced effect on the apoptosis rate. There are relatively few data on the proapoptotic activity of NSAIDs on human CRC cells. However, it has been noted that the two sulindac metabolites do have differential effects on apoptosis of SW480 human CRC cells (Piazza *et al*, 1997b). Recently, it has been reported that human colorectal mucosal apoptosis rates are not increased in NSAID users (Martin *et al*, 2002). This suggests that any proapoptotic effect of NSAIDs may also play only a minor role in their chemopreventative activity *in vivo*.

Although caspase-3 cleavage has been linked to sulindac-induced apoptosis of human CRC cells *in vitro* (Rice *et al*, 2003), assay of caspase-3 activity has not been performed previously in studies of the effect of NSAIDs on human CRC cells *in vitro*. There was a good correlation between induction of caspase activity and proapoptotic activity of individual NSAIDs, except for sulindac sulphide. Sulindac sulphide treatment is associated with cleavage of caspase-3 in HCT116 human CRC cells (Rice *et al*, 2003), which should increase caspase activity. However, sulindac sulphide treatment (300 *μ*M) was associated with decreased caspase-3/-7 activity in SW480 human CRC cells. Further investigation of whether sulindac sulphide has direct caspase inhibitory activity is now required.

Treatment with all NSAIDs, with the exception of rofecoxib, was associated with a reduction in total *β*-catenin protein levels of varying degrees (Table 2). Upregulation of *β*-catenin expression during colorectal carcinogenesis leads to accumulation of nuclear *β*-catenin, which is a prerequisite for increased CRT (Takahashi *et al*, 1998; Herter *et al*, 1999). Importantly, we observed that treatment with NSAIDs, except rofecoxib, was associated with a reduction in nuclear *β*-catenin content in SW480 human CRC cells. This is consistent with existing *in vitro* and *in vivo* data (Thompson *et al*, 2000; Rice *et al*, 2003; Boon *et al*, 2004), which includes the report of Brown *et al* (2001a), in which treatment with a similar panel of NSAIDs (indomethacin, sulindac, sulindac sulphone and meloxicam), but not celecoxib, was associated with decreased nuclear *β*-catenin staining in DMH-induced CRCs in rats. Although immunofluorescence for *β*-catenin protein does not easily allow quantitation of *β*-catenin levels, this technique may have more relevance to studies of the effects of NSAIDs on WNT signalling in human CRC cells than measurement of total cellular *β*-catenin levels.

The close relationship between the antiproliferative activity of individual NSAIDs and reduction in cyclin D1 protein levels in SW480 human CRC cells that we observed is in keeping with the known function of cyclin D1 as a cell cycle regulator of G1–S phase transition (Lukas *et al*, 1996). However, it has yet to be determined whether downregulation of cyclin D1 is a primary effect of NSAIDs or is secondary to G1 arrest induced by these drugs by another mechanism(s). Consistent with a central role for *cyclin D1* in cell cycle control, *cyclin D1* is a downstream target for several signalling pathways and has a complex promoter with multiple transcription factor binding elements (Lukas *et al*, 1996; Wang *et al*, 2001). *Cyclin D1* is a known *β*-catenin/TCF target gene (Shtutman *et al*, 1999). Therefore, one possible mechanism by which NSAIDs may downregulate cyclin D1 expression is by decreased CRT. Dihlmann and colleagues have reported that indomethacin and aspirin reduced CRT (measured using synthetic TCF-reporter genes, which included either a minimal *c-FOS* or SV40 promoter), as well as cyclin D1 mRNA and protein levels, in SW948 human CRC cells (Dihlmann *et al*, 2001). We have also previously demonstrated that indomethacin treatment is associated with reduction in cyclin D1 mRNA and protein levels in SW480 human CRC cells (Hawcroft *et al*, 2002). However, mutation of the single functional TCF-binding element in the *cyclin D1* promoter did not significantly abrogate the decrease in *cyclin D1* promoter activity induced by indomethacin, suggesting that other, CRT-independent mechanisms of action of indomethacin on the *cyclin D1* promoter are likely to exist (Hawcroft *et al*, 2002). Our observational data showing that there is no simple relationship between NSAID-induced changes in cyclin D1 protein levels and CRT (measured by TOPflash activity), despite reduction in nuclear *β*-catenin content, is consistent with the concept that reduced *cyclin D1* expression and inhibition of cell proliferation of human CRC cells occurs via a mechanism that is not simply explained by decreased CRT, subsequent to reduced *β*-catenin levels. This is perhaps not surprising given the complexity of control of cyclin D1 expression and the fact that NSAIDs are likely to have several different mechanisms of antineoplastic activity (Lukas *et al*, 1996; Hwang *et al*, 2002; Hull *et al*, 2003). Li *et al* have recently reported that sulindac sulphone induced a parallel decrease in *β*-catenin and cyclin D1 protein expression in SW480 human CRC cells (Li *et al*, 2002), building on an earlier report from the same group (Thompson *et al*, 2000). These authors overexpressed N-terminal mutant *β*-catenin protein in SW480 human CRC cells, which *partially* abrogated the exisulind-induced decrease in cyclin D1 protein and *partially* protected the cells from exisulind-induced apoptosis (Li *et al*, 2002). These data are also consistent with the concept that NSAIDs are likely to reduce cyclin D1 expression by CRT-dependent and -independent mechanisms. The conflicting data on the association between NSAID-induced changes in cyclin D1 expression and CRT suggest that cyclin D1 should only be used as a ‘read-out’ of CRT activity in human CRC cells with caution.

Our data on the effect of indomethacin on TOPflash activity conflict with those of Dihlmann *et al* (2001). However, our data on the effect of sulindac on TOPflash activity are similar to those recently reported by Boon *et al* (2004). Another study, which included experiments on the effect of sulindac on TOPflash activity in human CRC cells, reported that treatment of SW620 human CRC cells with a very high concentration (1600 *μ*M) of sulindac for 24 h was associated with increased ‘Tcf activity’ based on an increase in the TOPflash/FOPflash ratio (Bordonaro *et al*, 1999). Sulindac (10 *μ*M for 48 h) has been demonstrated to have no inhibitory effect on TOPflash activity in HEK293 cells (Orner *et al*, 2002) suggesting cell-type-specific activity of sulindac on CRT. Possible explanations for the discrepant data from experiments using human CRC cells include variable CRT-independent effects of NSAIDs on TOPflash constructs with different downstream promoters and/or variations in the methods used to analyse TOPflash and FOPflash activity data, which may not address adequately the issue of nonspecific effects of NSAID treatment on reporter gene expression.

In summary, NSAIDs have differential effects on *β*-catenin protein, cyclin D1 protein and CRT in human CRC cells. There is no simple relationship between alterations in total *β*-catenin protein levels and nuclear *β*-catenin localisation induced by NSAIDs and changes in CRT. Evidence is emerging that NSAIDs may also modulate WNT signalling via changes in the phosphorylation status of *β*-catenin and glycogen synthase kinase-3 (Dihlmann *et al*, 2003; Boon *et al*, 2004). More refined methods of testing the effects of NSAIDs on CRT in intestinal epithelial cells *in vivo* are now required in order to investigate further the activity of NSAIDs against this pivotal signal transduction mechanism in colorectal carcinogenesis.
